# Books Versus Screens: A Study of Australian Children’s Media Use During the COVID Pandemic

**DOI:** 10.1007/s12109-022-09899-w

**Published:** 2022-08-08

**Authors:** Sybil Nolan, Katherine Day, Wonsun Shin, Wilfred Yang Wang

**Affiliations:** School of Culture and Communications, The University of Melbourne, 3010 Victoria, Australia

**Keywords:** COVID pandemic, Children, Reading

## Abstract

As children’s use of screens increased during the COVID pandemic, their reading of traditional books was affected, a national survey of Australian parents shows. The study was conducted by researchers at the University of Melbourne to compare young people’s use of screens and books in the pandemic. Their online survey of 513 primary caregivers of children aged seven to thirteen around Australia showed that tablet use flourished during the pandemic and that COVID lockdowns influenced book buying and library borrowing in consequential ways for publishing and literature. Many parents believed their children’s use of screens had come at the expense of book reading.

## Introduction

When COVID-19 hit Australia in early 2020, lockdowns followed in most states, some lasting a few days and others for several months. Some state capitals experienced more than one long lockdown: Melbourne, the capital of Victoria, had the record for the most days in lockdown, cumulatively, of any city in the world [1]. At the height of the measures, most residents were unable to leave their homes except to travel short distances for essentials like food, daily exercise and medical care, there were nightly curfews, and interstate and international travel were banned.

The impact of longer lockdowns was just as hard on children as on adults. Many youngsters stayed home to learn at the kitchen table and missed out on playing with friends in the backyard or the schoolground. They also missed team sport, school trips and visits to the local pool, cinema and library. This resulted in more time in front of screens – an observation many parents made anecdotally, evidenced in news stories [2] and media surveys around the world [3, 4]. At the same time, tracking by Nielsen Bookscan provided intriguing signals that patterns of Australian reading were changing during the pandemic. In 2020, adult fiction sales surged 13.6% in value year on year, and the value of children’s book sales also increased, by 9.6% [5]. Nielsen commented that children’s books sales had benefited in 2020 from increased sales of study-at-home texts, as well as the continued success of the homegrown *Bluey* series [5, 6]. In 2021, while adult fiction once again grew, the value of children’s book sales fell marginally year on year [5].

Our research team, with expertise in both publishing and media studies, set out to investigate the impact of increased screen use during the pandemic on children’s reading for pleasure. We were particularly interested in traditional reading, i.e., of books printed on paper. Given that children were relying more on screens than before the pandemic and recurrent long lockdowns had been enforced in the two largest states, New South Wales and Victoria, we asked if the pandemic had disrupted young people’s reading and had perhaps even brought us to an inflection point at which many children’s use of screens would continue to increase at the expense of traditional book reading.

Realizing that lasting changes might emerge slowly, we decided to benchmark our research by an initial survey of Australian parents, while their memories of the COVID lockdown experience were still relatively fresh. The survey data would test our initial hypothesis that children’s book reading had changed during long lockdowns and would perhaps produce other insights we had not foreseen.

## Methodology

We designed a national online survey of 513 Australian parents (or other primary caregivers) of children aged between seven and thirteen, most of whom had been in lockdown within the past three months. In drafting the survey questions, we decided on a descriptive design that also allowed participants to elaborate on some of their responses [7]. In compiling the survey questions, we also sought to eliminate potential ambiguity by providing descriptions of specific terms, where needed, and avoid repetition and redundant data by testing the survey with a sample outside of the research group [8]. Doing so also helped us to envisage some clear objectives that might result in significant outcomes [9]. With a grant from our home department, we retained research service provider Qualtrics to recruit the survey participants in late October 2021, as long lockdowns in the largest state capitals, Sydney and Melbourne, ended and the Delta strain of Coronavirus receded. Almost ten per cent of the cohort were still in lockdown, 34% had been in lockdown within the past fortnight, and another 30% had been in lockdown within the past three months.

The majority of respondents were recruited from the states most affected by the pandemic, Victoria (31%) and New South Wales (30%), in line with the directions given to Qualtrics, but all states and mainland territories were represented. The sample included more female (64%) than male caregivers (36%), and eight out of ten (81%) respondents identified themselves as Australian. More than half (55%) were university-educated and from a household with more than $AUD100,000 annual income (i.e., well above the official national minimum wage of $AUD40,175) [10]. These income levels would have made purchasing discretionary items such as media devices and subscriptions possible for many families in the survey. The spread of age among the children of these respondents was quite even, while the gender split favoured boys (55%) over girls (45%). Half the respondents were instructed to answer in respect of their youngest child if they had more than one child aged seven to thirteen, the other half in respect of their eldest.

Statistical analyses were performed on the raw data. This included breaking the sample into a “lockdown” group who had been in lockdown any time within the past three months (n = 376, 73% of the total sample), and a “control” group who were outside that timeframe (n = 137, 27% of the total sample). There were two reasons for this: it seemed likely that more recent experiences of lockdown would result in more accurate answers and a higher degree of engagement with the survey; secondly, the control group would provide a meaningful measure of comparison within our data.

The survey asked about the types of media children were engaged with, by both device and genre. It then asked more questions about children’s reading, including where books were sourced from during lockdowns. We focused on books printed on paper, mainly because we were concerned that many parents could not have accurate awareness of children’s e-reading habits. This view was supported by the responses we received about media devices, as well as the methodology of another major Australian survey [3]. As we go on to discuss, tablets became prevalent during lockdowns, and were often used by children in their bedrooms. It would have been more difficult for parents to monitor what children were doing on their tablets than when they had a book in their hands.

We now turn to discussion of the major findings.

### Books and Tablets Both Popular for Children

One of the key questions we asked parents was what media devices children personally owned. Respondents ticked as many answers as applied in a checklist of possible devices which included books. Books were the most-owned, though tablets were not far behind (Table 1). Books and tablets were also the devices children were most likely to have in their bedrooms, though here there was clear daylight between the two.

**Table 1:** Which of the following devices does your child personally own? Which do they have in their bedroom? (n = 513)

Device	Personally own (%)	Have in their bedroom (%)
Books	66.5	66.5
Tablet	59.1	36.5
Smartphone	48.5	34.1
Video game console/player	44.6	26.9
Computer	39.8	30.0
TV set	25.0	26.9
e-reader	13.1	11.3
None of the above	2.3	6.8

**Fig. 1:**
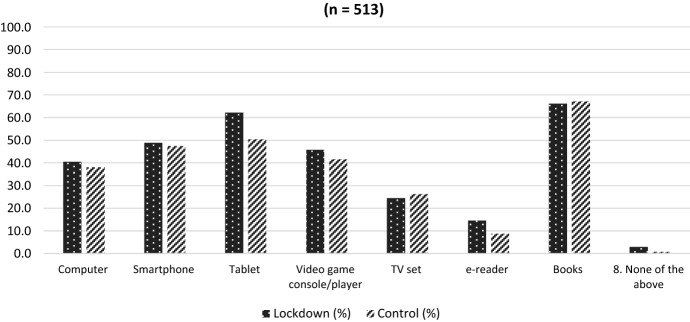
Personal ownership of media devices: Lockdown group versus control group (n = 513)

When these responses were analysed by group (lockdown group versus control group), the dominance of tablets as the preferred digital media device was clear: 62% of children in the lockdown group personally owned a tablet – only 4% less than the 66% who owned books. Long lockdowns clearly resulted in increased ownership by children of media technology (Fig. 1) and often meant increased use of it in their bedrooms. For example, 38% of children in the lockdown group had a tablet in their bedroom, compared with 33% in the control group.

E-readers were the device least likely to be owned by children. Among the lockdown group, the rate of ownership was somewhat higher yet only 15%.

### What Children Were Reading and How Much

The survey also investigated children’s reading habits before and during the lockdowns. It revealed that before the pandemic, 46% of respondents had to prompt their child to read and more than half (53%) of the children did not have a favourite book or author. These data suggest that a significant proportion of these children entered the pandemic without great attachment to reading for pleasure. The good news was that overall, 56% of parents said they regularly read a story to their child before the pandemic and the figure was 67% among parents of younger children aged seven to ten. Most of these parents continued this reading during lockdowns despite extra commitments to work and home schooling.

**Fig. 2:**
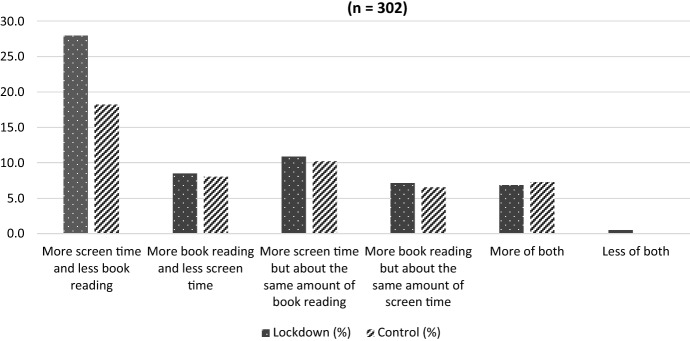
Comparison of change in relative use of screens and books: Lockdown group versus control group (n = 302)

Overall, respondents recorded that their child’s reading for pleasure was up by an average of 13 min a day. However, 59% of the sample (302 parents) also noticed a relative change in their child’s book reading and screen use during lockdowns. The nature of the change varied, but most frequently it was “more screen time and less book reading” (43%, or 130 parents of the 302 who had noticed a change). Comparing the lockdown and control groups, this trend was more clearly pronounced among the group who had been in lockdown recently (Fig. 2). More detailed interpretation of this data is in the discussion section below.

### Impact of Child Gender on Book Reading

Gender of child was fairly evenly covered in the survey with 281 respondents identifying their child as male, 232 as female and 0 as non-binary. The cross-tabulation of child gender and independent reading showed that during lockdown, both genders were reading slightly more overall for pleasure but girls read more than boys (7 h p/w for boys versus 8 h for girls). Boys also needed to be prompted to read more than girls.

The cross tabulation also showed that before lockdown, girls were more likely to engage in activities like craft, listening to music and watching movies/TV, and boys were more likely to engage in backyard play and computer games. The loss of opportunity for outdoor play with friends and for team sport may have hit boys harder, exposing them to increased reliance on computer games for recreation. This deserves further investigation.

While there was no substantial gender difference in reading genres, either before or during lockdowns, the top genres overall were picture books, chapter books (with and without illustrations) and school texts. Among boys, there was a trend during lockdowns away from picture books. Both younger and older boys read magazines and comics more (increasing 26 to 33% participation). This was perhaps because newsagencies, which sell everything from periodicals, books and stationery to lottery tickets and children’s toys, were considered essential businesses and many were still open during lockdowns. Overall, there was a 7% increase in younger children of both genders reading print magazines and comics. Children also read more e-books during lockdown, especially boys (a 5% increase to 23%).

### Reading and Age

Before lockdown younger children (aged 7–10, n = 277) predictably read more picture books (63%) and illustrated chapter books (47%) while older children (aged 11–13, n = 236) read chapter books with no illustrations (67%), and school texts (55%). During the lockdowns, however, the data revealed that the younger children actually read fewer picture books (down from 63 to 57%) and more chapter books (both illustrated and non-illustrated). Again, this could be partly linked to an increase in tablet use, which would be an area for further investigation. In terms of the screen to reading balance, there was consistency between the older and younger children groups: 43% of respondents who had younger children reported more screen time and less reading, and 44% who had older children reported more screen time and less reading.

### Where Books Were Sourced From

**Fig. 3:**
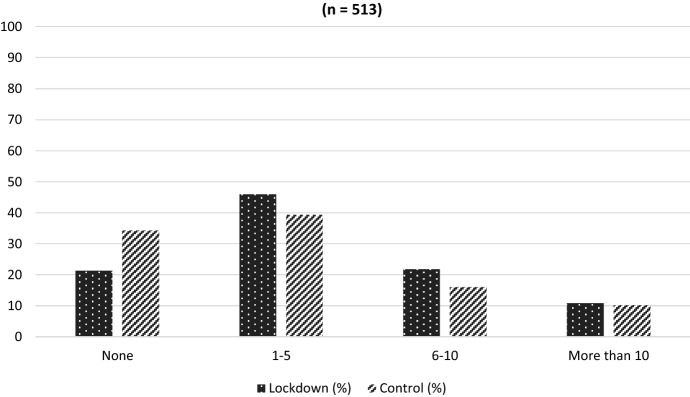
Number of books purchased for child during lockdown periods: Lockdown group versus control groups (n = 513)

This section considers book purchasing and library use during the lockdowns. A quarter of all parents in the survey reported that they had not bought a book for their child during the lockdowns, while 44% had bought between one and five books. These results could be a concerning indication for children’s reading, given that children caught up in longer lockdowns spent a lot of extra time at home. However, further statistical analysis of the lockdown and control groups tells a more encouraging story. Parents in the lockdown group were significantly more likely to have purchased books for their children than parents in the control group (Fig. 3).

The 386 parents who had bought one or more books for their children were then asked about where/how they had made the purchases. The biggest source was discount department stores (e.g., Target and Aldi). Online bookstores came a close second, while bricks-and-mortar bookstores came a distant third. This pattern was repeated closely in the breakdown of the lockdown and control groups (Table 2), suggesting consumers had a well-entrenched pattern of buying children’s books online and at less expensive prices. Further analysis within these groups showed that parents in the lockdown group who were from households earning more than $AUD100,000 a year were far more likely to head straight to an online bookstore (58%), while lower-income parents clearly preferred to do their online buying from discount department stores (51%).

**Table 2:** How did you purchase children’s books during lockdown? (%)

Source	Total (n = 386)*	Lockdown group (n = 296)	Control group (n = 90)
Online bookstore	54.9	44.10	33.60
Click and collect at local bookshop	22.3	18.10	13.10
Discount department store	59.3	47.30	37.20
Newsagency	17.9	14.10	11.70
Author’s own website	8.0	5.30	8.0
Book club (Scholastic)	16.6	14.10	8.0
Other	5.2	4.0	3.60

*Asked to those who reported they had purchased their child books during lockdowns

The sample were also asked to tick sources of information they consulted when deciding which children’s books to purchase during lockdowns (Table 3). For older children’s books, social media was the most common source (45%). For younger children’s books, word of mouth was the leading factor (38%). Teachers were also an important source of information about books, more so than librarians.

**Table 3:** How did you find out about these books during the lockdown periods? (%)

Source	Younger (n = 218)	Older (n = 168)
Word of mouth	38.1	41.7
Social media	34.9	44.6
Librarian	19.3	19.0
Child’s schoolfriend	22.0	28.6
Other parents	9.6	8.3
Relatives	15.1	25.0
Teacher	30.3	32.1
Grandparents	8.3	12.5
Television	14.2	15.5
Other	15.6	12.5

*Asked to those who reported they had purchased books for their child during lockdowns

**Fig. 4:**
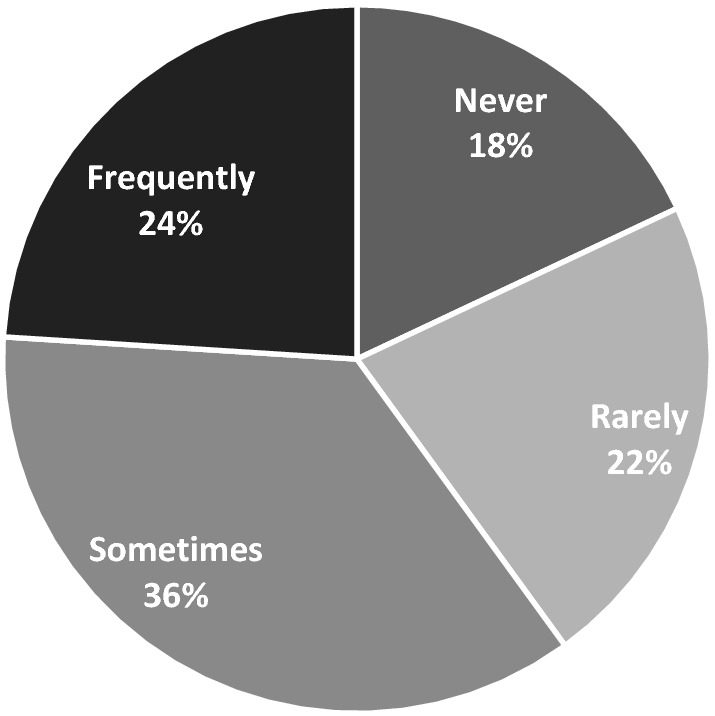
Frequency of library borrowing before lockdowns began (n = 513)

**Fig. 5:**
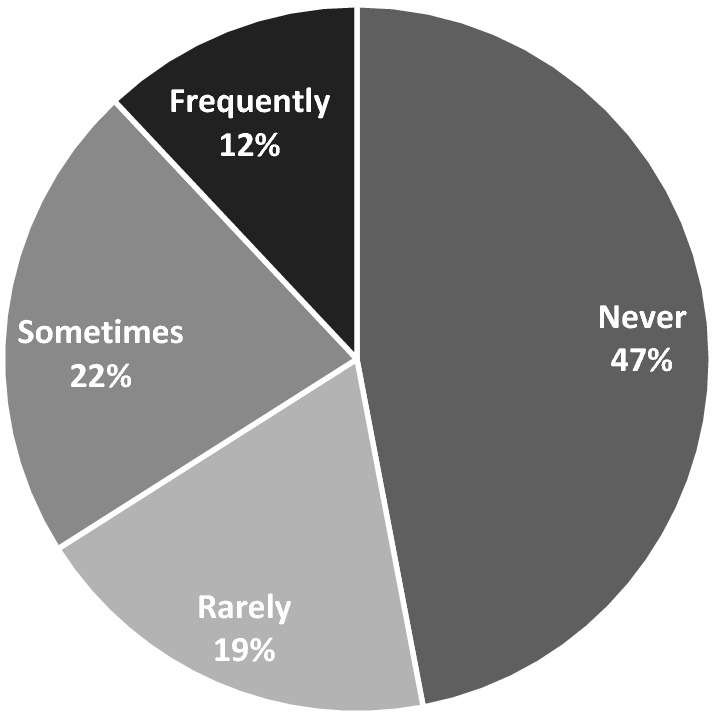
Frequency of library borrowing during lockdown periods (n = 513)

The picture for library borrowing during lockdowns was understandably grim: in the lockdown group, 36% “sometimes” borrowed a book for their child before lockdown, 24% frequently, 22% rarely and 18% never (Fig. 4). During lockdown, “never” jumped to 47% while only 12% frequently borrowed books for their children (Fig. 5). Significantly, though, we only asked about borrowing of physical books.

During lockdowns, not all libraries had a borrow and collect service. For example, of the 241 who answered that they “never” went to the library during lockdown, half said it was because their *school* library was closed. This shows a heavy reliance on schools to provide reading material for children.

## Further Discussion

The growing significance of tablets to children’s media use was the most important trend we observed in this survey. After books, tablets were the media device most owned by children; for children in lockdown, ownership of tablets and books was very closely aligned. This appears to be partly the result of home schooling, which in NSW and Victoria was mandatory during long lockdowns for all families that could undertake it. The Victorian government lent disadvantaged families more than 70,000 computers and tablets for educational purposes over the course of the pandemic, and then converted these loans to permanent ownership [11]; with more than one million school-age students in the state [12], tablet ownership was evidently important for families that could afford it. The pattern here was similar to that noted in other studies undertaken in 2021 [3, 4]: education at home was deeply implicated in increased screen use, which led to calls to revise national guidelines for safe screen use.

Our survey also revealed that across the board, children were using digital devices more during lockdowns. They spent more time on various digital devices after the COVID-19 outbreak began: 58% of respondents noted increased use of streaming services; 57%, both computers and tablets; 52%, video game consoles; and 51%, smartphones. More than 60% also said that their household had purchased a streaming media subscription during the lockdowns. There was a discernible difference (as shown in Fig. 1) between the lockdown group and the control group in media device ownership, particularly in relation to tablet ownership and video game consoles.

Lockdowns forced many families to become closer because they were unable to engage in social activities outside the family home, and this resulted in the growth of streaming subscriptions, which could be used by most or all family members, often together. Adults’ media use increased: 47% of parents reported an increase in screen media use, while 35% said they read books more often than before the pandemic. Parents’ media habits were being closely reflected to children of the same household during lockdowns; intergenerational media use was more clearly aligned for streaming media than for book reading.

The restrictions of lockdown were consequential for physical outlets for books, i.e., for bricks-and-mortar bookstores and libraries. Parents in the lockdown group bought more books for their children than parents in the control group, and clearly favoured online outlets over click-and-collect options at local bookstores. A quarter of respondents who had not bought a book for their child during lockdowns indicated that they thought bookshops were closed.

Fifty-nine respondents took the time to explain in writing why they had not bought books for their child during lockdown. Fifteen indicated that they were able to borrow from school or local libraries during the pandemic. Thirty-nine said ‘we have lots of books already’, or something similar. ‘We own 200 books and he [the child in question] was given a reading app from school full of more books,’ one caregiver wrote. ‘He has to read [the] books he has before I buy more to sit in a shelf,’ wrote a second. ‘He has soooo many books already,’ wrote a third. This set of answers suggested that children’s need for new books was finite. It could indicate emerging resistance to physical books among parents who were living at close quarters during the pandemic with children who preferred activities other than reading.

Remarkably, 257 adults who had observed changes in their children’s relative media usage during the pandemic agreed with the survey’s proposition that these changes were due to their child’s inability to see friends in person. This group constituted 50% of the total sample. As lockdowns decrease or disappear, and children are increasingly able to play together in person, it will be fascinating to see if parents and other caregivers report that their child’s use of screens has decreased.

## Conclusion

Our online survey of 513 parents around Australia concluded that many parents believed their children’s use of screens had come at the expense of book reading and that COVID lockdowns influenced book buying and library borrowing in consequential ways for publishing and literature. These findings were supported by the increasing dominance of tablets, the proliferation of other media devices during lockdowns, and that children could not engage in activities outside the home or play with friends. Our results showed that increased use of screens had certainly disrupted traditional reading: follow-up surveys will be required to determine if the reading habits of Australian children during and after the pandemic reached an inflection point at which their use of screens would continue to grow at the expense of traditional book reading.

The survey results will inform our research in 2022, which further investigates the frequency and specific constituents of children’s screen activity during the pandemic, particularly the role of e-books in children’s use of tablets. The findings have potentially significant consequences not only for children’s development but also for libraries and media industries, particularly book publishing and bookselling.

## References

[1] Enjoy your new freedoms, Melbourne, you’ve earned them [Editorial]. Melbourne, Australia: The Age. 2021 21 Oct. Available from: https://www.theage.com.au/national/victoria/enjoy-your-new-freedoms-melbourne-you-ve-earned-them-20211021-p591vv.html

[2] Geddes L, Marsh S. Concerns grow for children’s health as screen times soar during Covid crisis [Internet]. The Guardian. 2021 Jan 22. [Cited 2022 Mar 30] Available from: https://www.theguardian.com/world/2021/jan/22/children-health-screen-times-covid-crisis-sleep-eyesight-problems-digital-devices

[3] Arundell L, Veitch J, Sahlqvist S (2021). Changes in Families’ Leisure, Educational/Work and social screen time behaviours before and during COVID-19 in Australia: Findings from the our life at home study. Int J Environ Res Public Health.

[4] Eales L, Gillespie S, Alstat RA (2021). Children’s screen and problematic media use in the United States before and during the COVID-19 pandemic. Child Dev.

[5] Nielsen Bookscan (2022). Data and explanations supplied to authors.

[6] Brumm J (2018) *Bluey*. Brisbane, Australia: Ludo Studios; 2018-. The books are published by Penguin Random House in Australia, UK and other countries.

[7] Fink A (2003). The Survey Handbook.

[8] Patton MQ (2002). Qualitative Research Evaluation Methods.

[9] Fink A (1995). How to Sample in Surveys.

[10] Fair Work Ombudsman. Minimum wages [Internet]. Australian government; 2022 [cited 2022 Mar 31]. Available from: https://www.fairwork.gov.au/pay-and-wages/minimum-wages#national

[11] Victorian Government. Government responses to the recommendations of Public Accounts and Estimates Committee’s Inquiry into the Victorian Government’s response to the COVID-19 pandemic. Melbourne, Australia: Parliament of Victoria, 2021. https://www.parliament.vic.gov.au/file_uploads/Government_response_to_final_report_of_PAEC_s_Inquiry_into_the_Victorian_Government_s_response_to_the_COVID-19_pandemic_-_Final_for_tabling_zk158vfP.PDF

[12] Education and Training. Summary statistics for Victorian schools [Internet]. Victorian government; 2021 Jul [cited 2022 Mar 30]. Available from: https://www.education.vic.gov.au/Documents/about/department/brochurejuly.pdf

